# Nutrient Enrichment Mediates the Relationships of Soil Microbial Respiration with Climatic Factors in an Alpine Meadow

**DOI:** 10.1155/2015/617471

**Published:** 2015-08-12

**Authors:** Ning Zong, Jing Jiang, Peili Shi, Minghua Song, Zhenxi Shen, Xianzhou Zhang

**Affiliations:** ^1^Lhasa National Ecological Research Station, Key Laboratory of Ecosystem Network Observation and Modelling, Institute of Geographic Sciences and Natural Resources Research, Chinese Academy of Sciences, A11 Datun Road, Chaoyang District, Beijing 100101, China; ^2^University of Chinese Academy of Sciences, No. 19A Yuquan Road, Shijingshan District, Beijing 100049, China; ^3^Nanjing Agricultural Institute of Jiangsu Hilly Region, Nanjing, Jiangsu 210046, China; ^4^Key Laboratory of Ecosystem Network Observation and Modelling, Institute of Geographic Sciences and Natural Resources Research, Chinese Academy of Sciences, A11 Datun Road, Chaoyang District, Beijing 100101, China

## Abstract

Quantifying the effects of nutrient additions on soil microbial respiration (*R*_*m*_) and its contribution to soil respiration (*R*_*s*_) are of great importance for accurate assessment ecosystem carbon (C) flux. Nitrogen (N) addition either alone (coded as LN and HN) or in combination with phosphorus (P) (coded as LN + P and HN + P) were manipulated in a semiarid alpine meadow on the Tibetan Plateau since 2008. Either LN or HN did not affect *R*_*m*_, while LN + P enhanced *R*_*m*_ during peak growing periods, but HN + P did not affect *R*_*m*_. Nutrient addition also significantly affected *R*_*m*_/*R*_*s*_, and the correlations of *R*_*m*_/*R*_*s*_ with climatic factors varied with years. Soil water content (Sw) was the main factor controlling the variations of *R*_*m*_/*R*_*s*_. During the years with large rainfall variations, *R*_*m*_/*R*_*s*_ was negatively correlated with Sw, while, in years with even rainfall, *R*_*m*_/*R*_*s*_ was positively correlated with Sw. Meanwhile, in N + P treatments the controlling effects of climatic factors on *R*_*m*_/*R*_*s*_ were more significant than those in CK. Our results indicate that the sensitivity of soil microbes to climatic factors is regulated by nutrient enrichment. The divergent effects of Sw on *R*_*m*_/*R*_*s*_ suggest that precipitation distribution patterns are key factors controlling soil microbial activities and ecosystem C fluxes in semiarid alpine meadow ecosystems.

## 1. Introduction

Soil nutrient, especially nitrogen (N) availability, is an important factor determining primary productivity in many terrestrial ecosystems [1–3]. N enrichment by either N deposition or anthropogenic fertilization could enhance plant growth and promote net ecosystem primary productivity [4, 5]. Obviously, N enrichment can increase ecosystem carbon (C) sequestration by photosynthetic fixation on atmospheric carbon. However, the effect of N enrichment on CO_2_-C emissions from ecosystems, especially from the belowground portion, is inconsistent, being found to be positive [6–9], negative [10–12], or neutral [13–15]. Extensive research has focused on the effects of nutrient enrichment on soil respiration (*R*_*s*_), while the effects of nutrient enrichment on soil respiration (*R*_*m*_) and the contribution of *R*_*m*_ to *R*_*s*_ have received less attention. As a key component of *R*_*s*_, investigations on the effects of nutrient enrichment on soil microbial activity improve our understanding of potential effects of global change.

Principally, *R*_*s*_ consists of autotrophic respiration (primarily from roots) and heterotrophic respiration (mainly from soil microbes) [16]. Each of these components generally accounts for approximately 50% of the soil CO_2_ efflux [16–18]. However, this proportion varies dramatically in different ecosystems because root and soil microbial respiration differ in their sensitivity to changes of nutrient conditions [19, 20]. N enrichment probably either enhances root respiration by increasing fine root production [21, 22] or reduces root respiration by stimulating fine root turnover [23] while N amendment can either stimulate *R*_*m*_ by the increasing soil available N or reduce *R*_*m*_ due to the suppression on soil microbes after input of the high N amount [8, 24]. Therefore, accurately quantifying *R*_*m*_ can help us identify the source of variation in *R*_*s*_ [25, 26]. Meanwhile partitioning of *R*_*s*_ is essential to further detect the sensitivity of *R*_*s*_ and *R*_*m*_ to the increasing nutrient availability. Knowledge about the sensitivity is critical for accurately assessing the responses of belowground carbon fluxes to nutrient enrichment in the face of increasing of atmospheric N deposition, agricultural fertilization, and soil nutrient availability due to soil warming.

In the short term *R*_*m*_ is strongly and positively related to temperature [27, 28]. Exponential function has been widely used to describe the sensitivity of *R*_*m*_ to soil temperature. However, it has been proved that this relationship is regulated by soil water availability, especially in long-term and large scale [29]. In semiarid and arid region, precipitation is the main factor regulating plant growth and soil microbial activity [30, 31] due to the great contribution of precipitation to soil water availability [32]. Nutrient additions to soil can affect microbial activity [24, 33], yet it is not clear if these impacts can interact with the regulation effects of soil water availability.

The Tibetan Plateau covers about 2.5 million km^2^ with an average altitude of more than 4,000 m a.s.l. and 35% of that area is occupied by alpine meadows [34]. As the hinterland of the Tibetan Plateau, most of the meadows are located in the semiarid area. Most N is in organic form, since low temperature restricts decomposition of soil organic matter [35]. Therefore, plant growth and soil microbial activity are limited by the low soil N availability [7]. Moreover, phosphorus (P) is another essential element for plant growth and previous studies indicated that addition of N could induce P deficiency in grasslands [36]. N and P fertilization has been widely used as an efficient grassland management technique to recover degraded alpine meadow and to increase primary productivity to meet the needs of livestock [37, 38]. The N deposition rate in this area is about 7 kg N hm^−2^ yr^−1^ in the 2000s [39, 40] but is projected to increase to 40 kg N hm^−2^ yr^−1^ by 2050 [41]. In addition, the Tibetan Plateau is experiencing climatic warming [42, 43] and is predicted to experience “much greater than average” increases in surface temperature in the future [44] which mean that soil available nutrients may increase as the soil warming. Several lines of evidence have shown that the soil nutrient condition is improving in this meadow [29, 45]. Here we conducted a continuation of N and P addition study from 2008 and measured *R*_*s*_ and *R*_*m*_ during the growing season from 2010 to 2012. Our objectives were to (1) examine the effects of N and the combination of N and P on *R*_*m*_ and the contribution of *R*_*m*_ to *R*_*s*_ during growing seasons and (2) detect how nutrient enrichment regulates the correlations of *R*_*m*_ and *R*_*m*_/*R*_*s*_ in years with different variations in precipitation patterns. We hypothesize that (1) nutrient enrichment may decrease the contribution of *R*_*m*_ to *R*_*s*_ as belowground biomass was promoted by N + P treatments [46] and (2) the regulation effects of climatic factors on *R*_*m*_ may intensify as soil microbial biomass was high in N + P treatments [47].

## 2. Materials and Methods

### 2.1. Study Site

The study site is located in the midsouth portion of the Tibetan Plateau in the grassland station of Damxung County (91°05′E, 30°51′N, 4333 m a.s.l.). This site is characterized as semiarid continental climate influenced by monsoons from the Pacific Ocean. Mean annual temperature is 1.3°C with a minimum of −10.4°C in January and a maximum of 10.7°C in July. Difference between diurnal temperatures is 18.0°C. Annual precipitation is 477 mm, 85% of which is concentrated from June to August. The data given here were mean values based on climate data collected from 1962 to 2006 [48]. This experiment was conducted in an alpine meadow dominated by* Kobresia pygmaea*,* Stipa capillacea* and* Carex montis-everestii*, and about 15 species present in per square meter. The dominant species contribute over 40% of the aboveground biomass in this meadow. Vegetation cover ranges from 30% to 50% depending on yearly precipitation [48]. The soil is classified as Mat-Gryic Cambisol, corresponding to Gelic Cambisol, with a depth of about 0.3–0.5 m. Soil particle composition is 67.02% of sand, 18.24% of silt, and 14.74% of clay [49]. Detailed soil characteristics can be found in literature [50].

### 2.2. Experimental Design

An area of 40 m × 40 m alpine meadow with uniform vegetation cover was selected as the field fertilization experiment site. Twenty-five 5 m × 5 m split plots were laid out in a complete randomized block design with 5 replicates for each of the 5 treatments which included CK (i.e., no nutrient addition), 2 levels of N enrichment, and 2 levels of combinations of N and P enrichment. Plots were separated by 2-meter aisles as buffering zones. A study carried out in temperate grassland indicated that N addition rate more than 10.5 g N m^−2^ yr^−1^ did not affect plant production either in mature or in degraded grasslands [38]. Thus, two levels of N, 5 and 10 g N m^−2^ yr^−1^ (hereafter coded as LN for low N and HN for high N, resp.), were manipulated in the alpine meadow. Considering that alpine communities in this semiarid region are probably colimited by N and P availability [51, 52], we chose to combine constant 5 g P m^−2^ yr^−1^ with the LN and HN treatments (hereafter coded as LN + P and HN + P, resp.). Granular CO(NH_2_)_2_ and (NH_4_)_2_HPO_4_ fertilizers were directly applied before plant seedling establishment in each year since 2008 (June 15 in 2010, June 8 in 2011, and June 15 in 2012). Fertilizers were applied in the evening in fine weather to reduce leaching or volatilization. Plots were located in winter rangelands which meant no grazing from May to September and grazed in other months. For each treatment, four replicate plots were randomly chosen for measurements.

### 2.3. Field Sampling and Measurements

Root exclusion was used to estimate soil microbial respiration by comparing CO_2_ efflux rates from soil surfaces with and without living roots [16, 53]. A previous study in our meadow site showed that roots in the top 0–0.15 m of soil accounted for more than 95% of the total root biomass (0–0.5 m) [54]. Therefore, we created a root-free soil quadrat in each plot, with an area of 0.5 m × 0.5 m and depth of 0.15 m, by removing both aboveground and belowground plant materials in every 5-cm layer and backfilling the soil according to the original order [55, 56], in May before fertilization. Then one polyvinyl chloride (PVC) collar (20 cm in diameter and 15 cm in height) was placed on the plant-free quadrat in each plot. The PVC collars were inserted into the soil to a depth of 13 cm which prevented root growth in them but allowed the movement of water and soil microbes [57]. The quadrats with collars were kept free of seedlings and plant growth by frequent manual removal during the growing season. Thus, it was assumed that CO_2_ efflux measured within the collars was derived only from soil microbes and this respiration was coded as *R*_*m*_ [55]. Similar quadrats without root exclusion were also created in each plot and collars 5 cm in height were inserted into soil to a depth of about 3 cm. These collars retained intact plant root, and CO_2_ efflux measured in these collars was treated as *R*_*s*_. To minimize disturbance, the deep collars were installed one month before the first measurement and the aboveground plant material within the shallow collars was clipped to ground level and litter was removed 24 hours prior to each measurement [57]. A previous study in our meadow site indicated that daily mean values of *R*_*s*_ and *R*_*m*_ were quite close to the values measured between 09:00 and 11:00 a.m. [58]. Therefore, *R*_*s*_ and *R*_*m*_ were directly measured by a portable soil CO_2_ flux system (LI-8100, LI-COR Biosciences, Lincoln, NE, USA) between 09:00 and 11:00 a.m. on each measuring date. The interval between measurements was 10–15 days during the growing seasons from late June to late September in 2010, 2011, and 2012. We tried to select measuring dates at least two days after a rainfall event to avoid any pulse effect of precipitation on *R*_*s*_ and *R*_*m*_.

Soil temperature (°C) and volumetric soil moisture (m^3^ m^−3^) at 5 cm depth were collected from the nearby eddy covariance system (100 m away from our experimental site) automatically recording every 30 minutes. The representative range of this observation system is about 200 m [58].

### 2.4. Statistical Analysis

Repeated measures ANOVAs were used to analyze effects of nutrient fertilization and sampling year on *R*_*m*_ and *R*_*m*_/*R*_*s*_, with measuring date as the repeated variables. Since the effects of year and date and fertilization and the interactions between year and date on *R*_*s*_ and *R*_*m*_ were significant (*P* < 0.001), and the interactions between year and fertilization significantly affected *R*_*m*_ (*P* < 0.001, Table 1), we used a one-way ANOVA to test the differences in *R*_*m*_ and *R*_*m*_/*R*_*s*_ among fertilization treatments followed by Tukey's test for multiple comparisons in each year. Regression analyses were also used to test the correlations of *R*_*m*_/*R*_*s*_ with Sw and *T*_*s*_ both in CK and the combination of N and P treatments (LN + P, HN + P) in the three consecutive years, respectively. In these regression analyses, only N + P (combined treatments of LN + P and HN + P) and CK were concerned due to significant effects of nutrient addition on *R*_*m*_ occurring only in N + P treatments. All the analyses were performed in SPSS 16.0 (SPSS for Windows, version 16.0, Chicago, USA).

## 3. Results

### 3.1. Effects of Nutrient Enrichment on Seasonal Variations of *R*_*m*_

**Figure 1:**
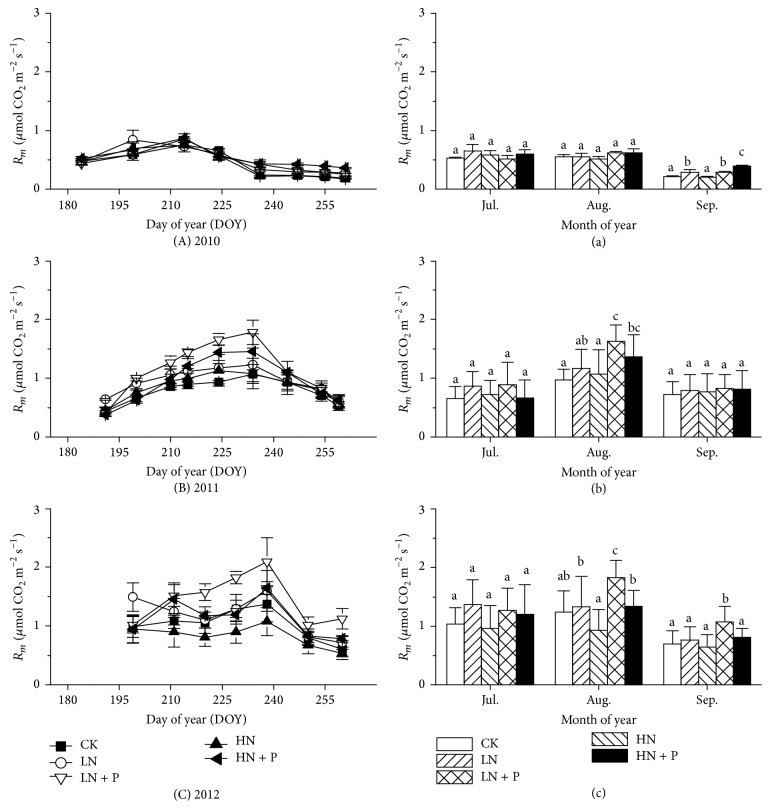
Seasonal variations (A, B, and C) and monthly mean values (a, b, and c) of soil microbial respiration during the 2010, 2011, and 2012 growing seasons. Bars sharing the same letters indicate no significant difference at* P* < 0.05 significant level. LN, HN, LN + P, and HN + P represent low N (5 g N m^−2^ yr^−1^), high N (10 g N m^−2^ yr^−1^), low N combined with P ((5 g N + 5 g P) m^−2^ yr^−1^), and high N combined with P ((10 g N + 5 g P) m^−2^ yr^−1^), respectively, and in the control treatment (CK) neither N nor P was added.

*R*_*m*_ showed significant seasonal variations (Figures 1(A), 1(B), and 1(C); Table 1,* P* < 0.001). LN or HN addition did not affect *R*_*m*_ relative to CK in each year of the experiment with the exception of higher *R*_*m*_ under LN treatment in September 2010 (Figure 1(a)). However, LN + P and HN + P enhanced *R*_*m*_ relative to CK in September 2010 (Figure 1(a)) and in August 2011 (Figure 1(b)). Similarly, LN + P increased *R*_*m*_ relative to CK in August and September of 2012 (Figure 1(c)).

### 3.2. Effect of Nutrient Enrichment on Contribution of *R*_*m*_ to *R*_*s*_

**Figure 2:**
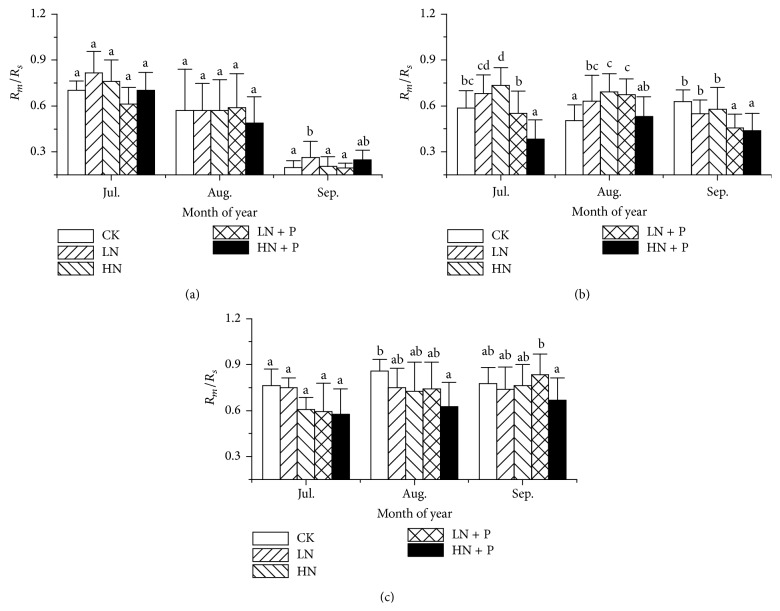
The contribution of soil microbial respiration (*R*_*m*_) to soil respiration (*R*_*s*_) in every July, August, and September during the 2010, 2011, and 2012 growing seasons in different fertilization treatments. See Figure 1 for abbreviations.

The contribution of *R*_*m*_ to *R*_*s*_ (*R*_*m*_/*R*_*s*_) varied with measured dates and years (Table 1,* P* < 0.001). In 2010, *R*_*m*_/*R*_*s*_ decreased sharply in September, while in 2011 and 2012 *R*_*m*_/*R*_*s*_ was relatively steady throughout the growing seasons (Figure 2). Nutrient enrichment also significantly affected *R*_*m*_/*R*_*s*_ but varied with years (Figure 2; Table 1,* P* < 0.001). In 2010, LN significantly increased *R*_*m*_/*R*_*s*_ in September, and HN increased it in July and August in 2011 (Figures 2(a) and 2(b)) while in 2011 HN + P decreased it compared with CK (Figures 2(a) and 2(b)), and this pattern also occurred in August in 2012 (Figure 2(c),* P* = 0.001). The average *R*_*m*_/*R*_*s*_ during the entire growing season under N + P and CK was 45% and 46.4% in 2010, 53% and 57.2% in 2011, and 73.5% and 80.6% in 2012.

### 3.3. Patterns of Precipitation, Soil Water Content, and Soil Temperature

**Figure 3:**
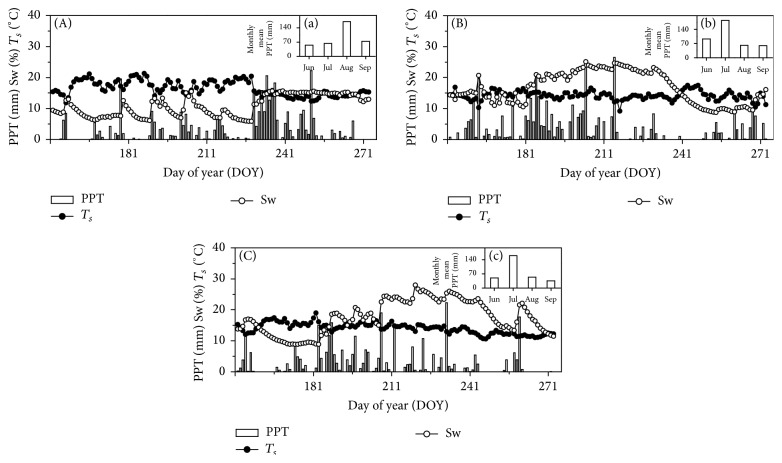
Daily precipitation (bars), daily mean soil temperature (line with solid circle), and moisture in 5 cm depth (line with open circle) from May to September in 2010 (A), 2011 (B), and 2012 (C). Precipitation in each month from June to September (in right up corner) was given in each year.

Total precipitation from June to September was 360.6, 397.1, and 299.6 mm during 2010, 2011, and 2012, respectively, and the distribution patterns differed among these three years (Figure 3). In 2010 high precipitation events mainly concentrated at the end of the growing season (from mid-August to early September) (Figure 3(A), (a)) whereas, in 2011 and 2012, high precipitation events synchronized with the peak plant growth (from July to early August, Figures 3(B) and 3(C)). Specifically, precipitation in July was 63.6, 183.9, and 159.9 mm in 2010, 2011, and 2012, respectively, which amounted to 17.5%, 46.3%, and 53.4% of total precipitation from June to September (Figures 3(a), 3(b), and 3(c)). Moreover, precipitation in August was 169.8, 61.5, and 54.2 mm in 2010, 2011, and 2012, respectively, amounting to 47.1%, 15.4%, and 18.1% of total precipitation from June to September (Figures 3(a), 3(b), and 3(c)). In 2010 Sw remained with very low values till August and then increased sharply as rainfall rising, but in 2011 and 2012 they were very high from July to August and then decreased in late growing season. All the variations in Sw corresponded to the patterns of precipitation.

### 3.4. Correlations of *R*_*m*_/*R*_*s*_ with *T*_*s*_ and Sw

**Figure 4:**
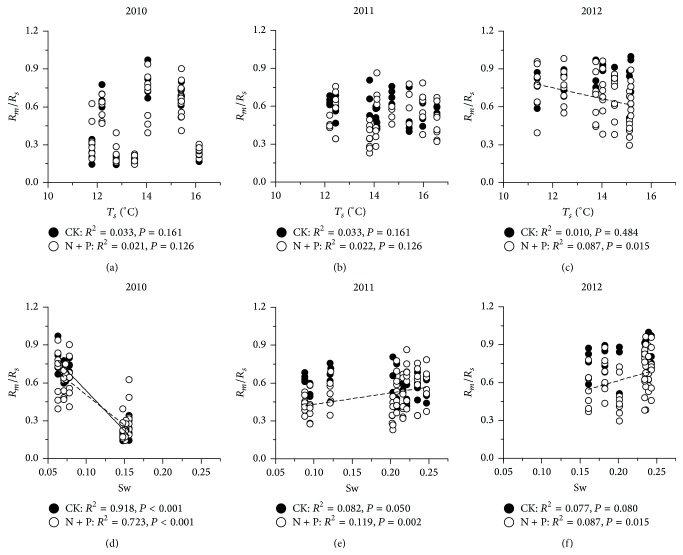
Relationships of contribution of soil microbial respiration (*R*_*m*_) to soil respiration (*R*_*s*_) with soil temperature and soil moisture in 5 cm depth under CK (soild circles) and combination of N and P treatments (including LN + P and HN + P treatments, open circles) in 2010, 2011, and 2012, respectively. See Figure 1 for abbreviations.

Correlations between *R*_*m*_/*R*_*s*_ and climatic factors showed that the variations of *R*_*m*_/*R*_*s*_ were mainly controlled by Sw, but these correlations differed with years (Figure 4). *R*_*m*_/*R*_*s*_ was negatively correlated with Sw in 2010 with larger variations of rainfall than the other two years (Figure 4(d), *R* ^2^ = 0.918,* P* < 0.001 in CK, *R* ^2^ = 0.723,* P* < 0.001 in N + P treatments) while in 2011 and 2012 *R*_*m*_/*R*_*s*_ was positively correlated with Sw, and these correlations were significant only in N + P treatments (Figures 4(e) and 4(f)). Soil temperature had little effects on *R*_*m*_/*R*_*s*_, and the correlation was only significant in N + P treatments in 2012 (Figure 4(c)).

## 4. Discussion

Our results showed that N addition alone at a rate greater than 5 g m^−2^ yr^−1^ did not affect *R*_*m*_ during the growing season from the third to the fifth experimental year (2010 to 2012) of nutrient enrichment. However, both LN + P and HN + P treatments increased *R*_*m*_ in most measuring dates, especially during the peak growth periods. Contrary to our assumption, LN + P did not decrease the contribution of *R*_*m*_ to *R*_*s*_ compared with CK. While consistent with the hypothesis, in N + P treatments the relationship between *R*_*m*_/*R*_*s*_ and Sw was more significant than in CK, and this relationship depended on precipitation distribution patterns. Our results indicate that the responses of *R*_*m*_/*R*_*s*_ to climatic factors were regulated by exogenous nutrient enrichment in this semiarid alpine meadow.

### 4.1. Effects of Nutrient Enrichment on *R*_*m*_ and the Contribution of *R*_*m*_ to *R*_*s*_

**Figure 5:**
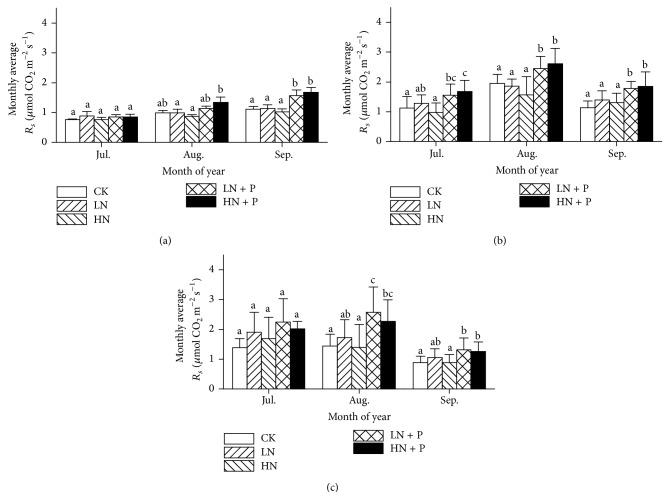
Monthly mean values of soil respiration during the 2010, 2011, and 2012 growing seasons. Bars sharing the same letters indicate no significant difference at* P* < 0.05 significant level. See Figure 1 for abbreviations.

Positive effects of N fertilization on *R*_*s*_ and *R*_*m*_ have been found in some terrestrial ecosystems [10, 59]. As N availability is often limited in most terrestrial ecosystems, exogenous N enrichment can stimulate soil microbial activity and thus enhance CO_2_ flux from soil. Another N fertilization experiment conducted in the same meadow in 2010 found N addition at rates of 1, 2, and 4 g N m^−2^ yr^−1^ significantly increased plant aboveground biomass, *R*_*s*_, and *R*_*m*_ [60]. However, in our N fertilization experiment adding N at the rate 10 g m^−2^ yr^−1^ did not significantly affect *R*_*s*_ and *R*_*m*_ (Figures 1 and 5). Additionally, N addition at the rate of 5 g m^−2^ yr^−1^ in this meadow did not significantly affect plant aboveground biomass (Table 2). Therefore, we presume that 5 g N m^−2^ yr^−1^ could be the saturation threshold for this alpine meadow ecosystem. Previous studies also found that *R*_*s*_ ceased to continue to increase after years of fertilization [61, 62]. The neutral or suppression of *R*_*s*_ and *R*_*m*_ under long-term and high N addition treatments could be due to labile C depletion [63, 64], reduction of microbial biomass [64, 65], inhibition of microbial activity [66], and/or reduction of the belowground allocation [59]. Nitrogen additions significantly accelerated decomposition of light soil carbon fractions, which caused the depletion of labile C [63]. High rates of N addition could also lead to toxicity and reduction to plants and soil microbes by soil acidification, which resulted from the depletion of base cations and the release of ammonium from the soil [67, 68]. In addition, increased N availability may reduce the investment of photosynthetic labile carbon to root systems and subsequently decreased the supply of labile carbon to soil microbes [24]. In our study, although N addition at 10 g m^−2^ yr^−1^ did not significantly decrease *R*_*s*_ and *R*_*m*_, the soil microbial biomass carbon indeed was much lower in HN than in CK (data were not presented). We presume that HN would decrease *R*_*m*_ if we continue HN addition in the coming years.

In our study LN + P enhanced *R*_*m*_ at the end of 2010 and in the middle of 2011 and 2012. Previous nutrient amendment studies showed that N availability was the main limitation of primary production in dry alpine meadows, while N and P availability colimited production in a wet alpine meadow in Colorado, USA [51, 69]. Another N and P fertilization experiment conducted in New Zealand showed that N addition changed the composition and activity of soil microbes, but the addition of P alone did not [70]. Additionally, it was found that P addition typically increased microbial biomass in the short term but generally decreased biomass over longer terms [71]. P addition alone was not concerned in our experiment and we failed to know if the positive effect derived from N + P or P addition alone. Therefore, the critical next step is to carry out P addition treatment in order to examine whether P limits plant growth and soil microbe activity in this meadow and to help in our understanding of the effects of N + P.

Our study showed *R*_*m*_/*R*_*s*_ was 46.4%, 57.2%, and 80.6% in CK during the 2010, 2011, and 2012 growing seasons, respectively, which are right within the range of 10%–90% across different ecosystems [16] and within the range of 35%–90% in a tallgrass prairie ecosystem over many years [72]. Generally, *R*_*m*_/*R*_*s*_ exhibits interannual variations with changes in climate and relevant physiological and ecological processes year by year [72–74]. Contrary to our assumption, LN + P treatments did not decrease the contribution of *R*_*m*_ to *R*_*s*_, while, in some sampling months, the effects of HN + P partly conformed to the assumption. Our previous study demonstrated that LN + P significantly enhanced belowground biomass in September [46]. It is well known that belowground biomass is the direct source of root respiration and this increase could enhance the contribution of root respiration to *R*_*s*_. However, the supply of liable carbon to soil microbes is mainly from root rhizodeposits and exogenous nutrient enrichment could stimulate soil microbial activities [24, 33]. Therefore, the contribution of *R*_*m*_ to *R*_*s*_ lies in the effects of nutrient enrichment on belowground biomass and soil microbial activities. In this study, LN + P treatment did not decrease the contribution of *R*_*m*_ to *R*_*s*_, indicating that nutrient enrichment has a greater impact on soil microbial activities than belowground biomass. However, in some sampling months HN + P decreased *R*_*m*_/*R*_*s*_ relative to CK and we infer that high N addition could inhibit soil microbial activity.

### 4.2. Effects of Soil Temperature and Moisture on *R*_*m*_/*R*_*s*_

CO_2_ emission from soil is regulated by several abiotic and biotic factors [53], such as temperature, rainfall events, soil moisture, soil physiochemical properties, and plant and soil microbial activities. In our study, the significant correlation between *R*_*m*_/*R*_*s*_ and Sw during growing season suggests soil water availability is an important factor limiting ecosystem C fluxes in this semiarid alpine meadow. However, differential responses of *R*_*m*_/*R*_*s*_ to soil water availability were also observed in these three years. The relationship between *R*_*m*_/*R*_*s*_ and Sw was negative in 2010 but positive in 2011 and 2012. This divergent correlation among years was attributed to the different precipitation patterns. Temperature varies usually in synchrony with precipitation during the growing season in this region; that is, high temperature and high precipitation corresponded to occurrence at the peak plant growth period, such as in 2011 and 2012, while high precipitation events mainly concentrated at the end of the growing season in 2010 owing to the late monsoon from the Pacific Ocean. For instance, 17.5% of total precipitation in July 2010 is far less than 46.3% and 53.4% of total precipitation in July 2011 and 2012, respectively. The continual and sharp increasing precipitation led to increase of Sw but decrease of *T*_*s*_ in August 2010, which led to low soil microbial activities. Correspondingly, *R*_*m*_/*R*_*s*_ maintained high levels from July to mid-August and decreased gradually from then on (Figure 2). Therefore, the asynchronous variations of *T*_*s*_ and precipitation accounted for the negative correlation between *R*_*m*_/*R*_*s*_ and Sw in 2010. However, in 2011 and 2012 with even distribution of precipitation during growing seasons, *R*_*m*_/*R*_*s*_ was positively correlated with Sw. The divergent effects of Sw on the contribution of *R*_*m*_ to *R*_*s*_ in years with different precipitation patterns suggest that precipitation distribution patterns are the key factors controlling soil microbial activities and ecosystem C fluxes in semiarid alpine meadow ecosystems.

As N availability is limited in alpine meadow ecosystems [35, 51], exogenous nutrient enrichment can stimulate soil microbial activities, and this has been proved in many previous studies [46, 75]. Although we did not measure soil microbial activities in this experiment, soil microbial biomass carbon was higher in N + P treatments than that in CK, especially in LN + P treatments [47], indicating that soil microbial activities were stimulated by exogenous nutrient enrichment. In 2011 and 2012 with even distribution of precipitation during growing seasons, the variations of *R*_*m*_/*R*_*s*_ did not depend on soil water availability in CK, while, in the N + P treatments with stimulated soil microbial activities, the variations of *R*_*m*_/*R*_*s*_ depended on soil water availability. This result indicated that nutrient enrichment mediates the relationships between soil microbial respiration and climatic factors through the stimulation of soil microbial activities.

## 5. Conclusions

N addition at a rate greater than 5 g N m^−2^ yr^−1^ did not significantly affect *R*_*m*_ and plant aboveground biomass; we presume that 5 g N m^−2^ yr^−1^ could be the saturation threshold for this alpine meadow ecosystem. During the years with large variations of rainfall, *R*_*m*_/*R*_*s*_ was negatively correlated with Sw, while, in years with even distribution of rainfall, *R*_*m*_/*R*_*s*_ was positively correlated with Sw. The divergent effects of Sw on the contribution of *R*_*m*_ to *R*_*s*_ in years with different precipitation patterns suggest that precipitation distribution patterns are the key factors controlling soil microbial activities and ecosystem C fluxes in semiarid alpine meadow ecosystems. In the future climate change scenarios, spatial and temporal changes in precipitation patterns may have great impacts on semiarid alpine meadow ecosystems. Meanwhile, our results also indicate that the increase of exogenous N deposition in the future climate change scenarios may mediate the controlling effects of climatic factors on *R*_*m*_/*R*_*s*_.

## Figures and Tables

**Table 1:** Results (*F* and *P* values) of repeated measures ANOVAs on the effects of nutrient fertilization (F) and year (Y) on soil microbial respiration (*R*_*m*_) and the contribution of *R*_*m*_ to *R*_*s*_ (*R*_*m*_/*R*_*s*_), with measuring date (D) as repeated variables, respectively.

	df	*R*_*m*_		*R*_*m*_/*R*_*s*_	
		*F*	*P*	*F*	*P*
Year (Y)	2	216.17	<0.001	57.44	<0.001
Date (D)	5	8.22	<0.001	79.81	<0.001
Fertilization (F)	4	14.70	<0.001	11.57	<0.001
Y × D	10	12.93	<0.001	72.77	<0.001
Y × F	8	2.87	0.004	2.84	0.006
D × F	20	1.43	0.11	2.69	0.010
Y × D × F	40	0.81	0.78	2.92	<0.001

**Table 2:** Monthly means (data in the parentheses represent the SE, *N* = 4) of aboveground biomass (AGB) in 2010, 2011, and 2012. Different letters in the same month within the same year indicate significant difference (*P* < 0.05) in monthly averages among treatments. See Figure 1 for abbreviations.

		CK	LN	HN	LN + P	HN + P
2010	Jul.	65.3 (6.4)^a^	66.0 (19.5)^a^	65.8 (16.6)^a^	87.6 (18.3)^b^	65.7 (16.2)^a^
	Aug.	89.7 (18.8)^a^	108.9 (28.3)^ab^	91.4 (14.3)^a^	130.2 (34.1)^bc^	141.9 (43.1)^c^
	Sep.	94.7 (33.8)^a^	103.1 (31.4)^a^	82.3 (20.6)^a^	136.9 (22.9)^b^	131.8 (28.8)^b^

2011	Jul.	38.2 (12.9)^a^	44.4 (19.6)^ab^	30.8 (12.1)^a^	72.6 (16.1)^c^	56.5 (16.1)^b^
	Aug.	81.8 (28.7)^a^	116.6 (53.8)^ab^	77.6 (30.3)^a^	159.8 (66.9)^b^	151.3 (75.1)^b^
	Sep.	81.6 (30.5)^a^	101.4 (32.3)^ab^	80.1 (22.0)^a^	136.2 (58.7)^b^	129.0 (43.8)^b^

2012	Jul.	55.5 (11.3)^b^	54.0 (17.6)^b^	37.2 (19.2)^a^	102.0 (24.7)^c^	73.6 (21.0)^c^
	Aug.	157.7 (86.6)^a^	182.9 (39.4)^a^	149.7 (69.1)^a^	281.9 (74.9)^b^	326.2 (84.0)^b^
	Sep.	91.8 (29.7)^a^	91.7 (30.5)^a^	101.6 (22.0)^a^	180.9 (46.1)^b^	204.3 (50.8)^b^
